# Evaluation of the effects of sagging shifts on isocenter accuracy and image quality of cone‐beam CT from kV on‐board imagers^*^

**DOI:** 10.1120/jacmp.v10i3.2930

**Published:** 2009-07-17

**Authors:** Imad Ali, Salahuddin Ahmad

**Affiliations:** ^1^ Department of Radiation Oncology Oklahoma University Health Sciences Center Oklahoma City OK 73104 USA

**Keywords:** sagging shifts, cone‐beam CT (CBCT), on‐board imager (OBI), isocenter, image quality

## Abstract

To investigate the effects of sagging shifts of three on‐board kV imaging systems (OBI) on the isocenter positioning accuracy and image quality of cone‐beam CT (CBCT). A cubical phantom having a metal marker in the center that can be aligned with the radiation isocenter was used to measure sagging shifts and their variation with gantry angle on three Varian linacs with kV on‐board imaging systems. A marker‐tracking algorithm was applied to detect the shadow of the metal marker and localize its center in the two‐dimensional cone‐beam radiographic projections. This tracking algorithm is based on finding the position of maximum cross‐correlation between a region‐of‐interest from a template image (including the metal marker) and the projections containing the shadow of the metal marker. Sagging shifts were corrected by mapping the center of the metal marker to a reference position for all projections acquired over a full gantry rotation (0–360°). The sag‐corrected radiographic projections were then used to reconstruct CBCT using Feldkamp back‐projection. A standard quality assurance phantom was used to evaluate the image quality of CBCT before and after sagging correction. Sagging affects both the positioning accuracy of the OBI isocenter and the CBCT image quality. For example, on one linac, the position of the marker on the cone‐beam radiographic projections depends on the angular view and has maximal shifts of about 2 mm along the imager x‐direction (patient's cross‐plane). Sagging produces systematic shifts of the OBI isocenter as large as 1 mm posterior and 1 mm left in patient coordinates relative to the radiation isocenter. Further, it causes spatial distortion and blurring in CBCT image reconstructed from radiographic projections that are not corrected for OBI sagging. CBCT numbers vary by about 1% in full‐fan scans and up to 3.5% in half‐fan scans because of sagging. In order to achieve better localization accuracy in image‐guided radiation therapy, sagging shifts of the kV OBI need to be corrected. In addition, correction of sagging improves image and provides better visualization of internal structures. Frequent quality assurance is required to monitor and maintain standards of variations in the mechanical accuracy of isocenter and image quality of CBCT because of sagging shifts.

PACS number: 87.57.Q, 87.57.cp

## I. INTRODUCTION

Image‐guided radiation therapy (IGRT)^(^ ^1^ ^–^ ^3^ ^)^ is an emerging technology that has the potential to enhance the outcome of advanced treatment techniques such as intensity‐modulated radiation therapy (IMRT),^(4)^ stereotactic radiation therapy^(^ ^5^ ^,^ ^6^ ^)^ and brachytherapy,^(^ ^7^ ^,^ ^8^ ^)^ where accurate patient setup and tumor localization are needed because of high‐dose gradients between tumor and critical structures. On‐board kV cone‐beam imaging systems provide volumetric CT imaging data of the target and surrounding anatomical structures such as bones and soft tissue. This system is superior to MV cone‐beam CT^(^ ^9^ ^–^ ^12^ ^)^ in terms of soft tissue visibility and image quality; however, it is inferior to conventional CT simulators.^(^ ^1^ ^,^ ^13^ ^,^ ^14^ ^)^ The three‐dimensional (3D) kV cone‐beam CT (CBCT) imaging data is currently used in IGRT for patient setup, tumor visualization, and localization^(^ ^1^ ^,^ ^3^ ^)^ just before or during treatment session. The on‐line current imaging data has the advantage of minimizing patient setup uncertainty by localizing the actual internal target instead of using external skin markers. Errors due to positioning shifts of the target between simulation and dose delivery are reduced by IGRT using updated real‐time volumetric CBCT data of the internal patient anatomy. Initial treatment plans can be modified by incorporating variation in patient anatomy due to organ motion, tumor deformation or growth, or fillings of critical structures by using 3D‐CBCT with valid tissue electron density for dose calculation. This may allow the use of adaptive radiation therapy (ART), which requires real time internal anatomy 3D imaging data, plan modification based on updated visualization and localization of the tumor and critical structures, and dose calculation using valid CBCT numbers.

Despite the clinical potential of CBCT imaging for IGRT and ART, this technique is limited by drawbacks of mechanical stability and image quality. Mechanical stability of the OBI isocenter is influenced by extension and retraction processes of the non‐rigid robotic arms of the source and imager, and sagging shifts in the rotation process of the heavy linac gantry. Other motions of the OBI include translational shifts to perform full‐fan (FF) or half‐fan (HF) scans in which the imager is centered or shifted towards one side of the patient to obtain small and large volume scans, respectively. The accuracy of the position of CBCT isocenter determines the accuracy of IGRT patient setup. Precise isocenter match of the on‐board imaging (OBI) system with the radiation isocenter and the planning CT isocenter is necessary for accurate patient setup and tumor localization. Offsets due to sagging^(15)^ or mismatch of radiation and imaging isocenters need to be corrected either using software or hardware tools. Further, the mechanical instability of the on‐board imager^(^ ^15^ ^,^ ^16^ ^)^ produces image artifacts in CBCT. Image artifacts and invalid CBCT numbers may limit CBCT use for IGRT and ART where superior soft tissue visibility and accurate tissue electron density are required for patient setup and treatment planning, respectively.

IGRT patient setup using internal patient anatomy from 3D‐CBCT^(^ ^1^ ^,^ ^3^ ^,^ ^17^ ^)^ is performed using different image registration techniques.^(18)^ These include registration of bony anatomy between the CT simulation and CBCT data, using fiducial markers, or registration on soft tissue using contours outlined in the planning process and projecting them on the CBCT slices. OBI sagging will limit the accuracy of image registration between CBCT and conventional CT used for treatment planning because of systematic errors in CBCT isocenter and image artifacts. Suboptimal image quality due to sagging produces miss‐judgment of the location of the target, omission of target volume from treatment coverage, or inclusion of normal tissue in the treatment. Accurate CT numbers and electron density of the tumor and surrounding structures are necessary for accurate dose calculation in adaptive radiation therapy. To minimize the effects of sagging, quality assurance procedures should be performed on a regular basis to test mechanical accuracy and image quality for the OBI.^(16)^

In this work, we have investigated the effect of sagging on the mechanical stability and image quality of three kV on‐board cone‐beam imaging systems. Systematic errors in positioning of OBI isocenter and CBCT imaging artifacts were measured and quantified. Software tools were developed to preprocess the cone‐beam projections and correct sagging effects in reconstructed CBCT. A quality assurance (QA) procedure was developed in our clinic for all the linacs with kV on‐board imaging to test regularly both mechanical stability and image quality due to sagging.

## II. MATERIALS AND METHODS

### A.1 On‐board imager

The mechanical stability and image quality were evaluated for three kV on‐board cone beam imagers that are mounted on two 2100EX and one Trilogy linacs (Varian Medical Systems, Palo Alto, CA). The OBI consists of a diagnostic quality kV X‐ray source and an amorphous‐silicon fat‐panel imager (PaxScan 4030CB) held by gantry‐mounted robotic arms. The effective imaging area of the fat panel imager is about $40\times 30\;{\text{cm}}^{2}$. The imager is operated in 2×2 binning mode with radiographic projections of full resolution in a matrix of 2048×1572pixels to reconstruct 3D volumetric CBCT. CBCT represented in this work was reconstructed from a 1024×7682D radiographic projections with a corresponding pixel size of $0.39\times 0.39\;{\text{mm}}^{2}$.^(19)^

CBCT scans are acquired by extension of the source and imager using the robotic arms, where the source and imager are setting opposite to each other at 100 cm and 50 cm from isocenter, respectively. Then the OBI system rotates around the imaged object in a full circle (360°) with the OBI always at 90° from MV beam. One scan takes nearly one minute for a full circle gantry rotation, during which time about 650 radiographic projections are acquired. The OBI cone‐beam system has two scanning modes: (a) FF with a maximum field of view (FOV) of 17 cm thickness and 25 cm diameter, and (b) HF with a FOV as large as 15 cm patient thickness and 50 cm diameter. FF and HF scans can be combined with and without head and body bowtie filters. The bowtie filter (aluminum $2.8\;g/{\text{cm}}^{3}$) has variable thickness that decreases near the center of the beam to compensate for radiation absorption by the thick part of the phantom. Other advantages of scanning with the bowtie filters include reduction of patient dose and image noise by removing low energy scattered photons from the head of the imager. CBCT scanning protocols use various kV, mA, and ms to obtain appropriate image quality. For example, 125 kV, 80 mA and 25 ms were used for scans with bowtie filter, and 125 kV, 80 mA and 8 ms were used for scans without bowtie filter.

### A.2 Phantoms

#### A.2.1 Cubical phantom and OBI mechanical stability

A cubical phantom was used to measure sagging shifts and evaluate the mechanical stability of the OBI system. This cubical phantom is a $5\times 5\times 5\;{\text{cm}}^{3}$ cube (Varian Medical Systems, Palo Alto, CA) that has a spherical metal marker with a diameter of 2 mm placed in the center of the phantom. The center of the cubical phantom is aligned with radiation isocenter by aligning the room lasers with crosshair markers on the phantom surface. These markers allow alignment of the phantom with the side (vertical and horizontal) and sagittal lasers. On our linacs, the room lasers reproduce the position of the radiation isocenter within 0.5 mm in the different directions. The room lasers are aligned with the radiation isocenter using the Winston and Lutz procedure.^(20)^ This includes alignment of a pointer phantom with the radiation isocenter. Several radiographic projections for small fields (1 cm^2^) that are defined with the multileaf collimator are acquired at different gantry angles and using the portal imager. The position of the pointer phantom is adjusted to within 0.5 from the center the field and this represents the accuracy of the alignment of the metal marker with the radiation isocenter. Then, the lasers are aligned with the crosshairs on the pointer phantom. The machining accuracy of the cubical phantom was tested by film radiographic imaging and found that the metal marker is located in the center of the phantom and aligned with the crosshairs on its surface within 0.1 mm.

#### A.2.2 Catphan phantom and CBCT image quality

A commercially available phantom, Catphan 500 (The Phantom Laboratory, Inc., Salem, NY)^(21)^ was used to evaluate image quality parameters of the CBCT. This phantom is cylindrical with a diameter of 20 cm and a length of 20 cm. It has different modules that include: (a) an image uniformity module (CTP 486) to evaluate CT number uniformity and noise, (b) a linearity module (CTP 404) to evaluate quantitatively CT sensitivity, (c) a spatial resolution module (CTP 528) to evaluate high‐contrast resolution and spatial resolution, and (d) a low‐contrast resolution module (CTP 515) to evaluate ability to resolve low‐contrast level. The phantom was aligned for scanning with the room lasers using fiducial markers on both sides and top of the phantom. Then, it was scanned using both HF and FF protocols, and CBCT was reconstructed from radiographic projections before and after correction for sagging shifts.

**Figure 1:**
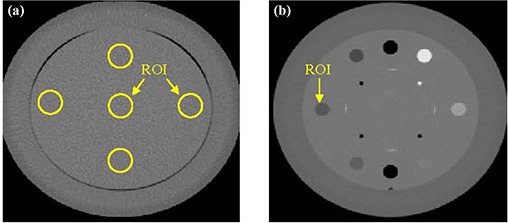
CBCT slices that represent: (a) the uniformity module with regions‐of‐interest (ROI) used to calculate average CT and standard deviation; (b) the linearity module.

The sagging effect on CT number uniformity was measured by cropping regions‐of‐interest (ROI) from different positions: center, right, left, anterior, and posterior of a uniformity CBCT slice (module CTP 486) reconstructed from projections before and after correction for sagging shifts, as shown in Fig. 1(a). Mean CT number was calculated over approximately 400 pixels for each ROI. The CT number dependence on the OBI sagging was determined by calculating mean CT over a ROI (100 pixels) derived from a slice, as shown in Fig. 1(b), which is made from different materials (cylindrical rods with diameter=1cm,length=3cm) such as Tefon, Derlin, Acrylic, low density polyethylene (LDPE), PMD, and air from a linearity CBCT slice (Fig. 1(b)). Image noise was calculated from normalized standard deviation of CT numbers, $\gamma =\frac{\sigma }{<CT>}$, in a sample of about 400 pixels from a CBCT slice reconstructed from cone‐beam radiographic projections before and after correction for OBI sagging. Here, σ is the standard deviation of CT numbers (in Hounsfield units) and <CT> is the average CT number over all pixels from the corresponding ROI.

### A.3 Sagging shifts of the OBI

**Figure 2:**
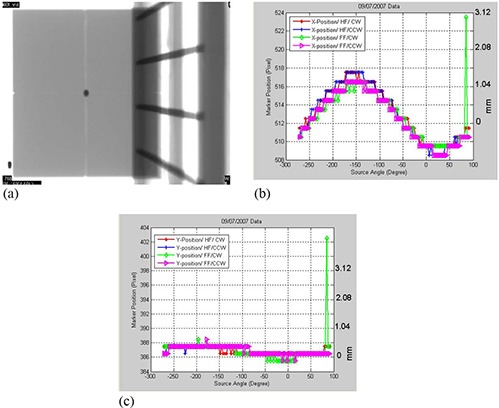
(a) A cone‐beam radiographic projection where the dark region represents the shadow of the metal marker in the center of the phantom. 2(b) Position shifts of the metal marker in the projections along the x‐axis as a function of view angle, θ, for the CBCT scanning protocols that include half‐fan scan with the gantry rotated clockwise (HF/CW), half‐fan and counter‐clockwise gantry rotation (HF/CCW), and full‐fan scans in which the gantry was rotated clockwise (FF/CW) and counter‐clockwise (FF/CCW), respectively. 2(c) Marker position along the y‐axis (superior‐inferior direction) as a function of gantry angle for the scanning protocols as in Fig. 2(b) for a 21 EX machine with a kV on‐board imager.

The OBI sagging shifts were measured using the cubical phantom, which was scanned using a combination of CBCT scanning protocols such as HF and FF with and without bowtie filters. The metal marker centered inside the cubical phantom creates a dark shadow in the radiographic projections (Fig. 2(a)), and has a circular shape and low intensity. The position of the shadow of the metal marker in the projections represents the position of the OBI isocenter, which should be projected to the same point on the 2D imager for the different angular views assuming that the OBI is a rigid body. However, the OBI robotic arms of the imager and the radiation source sag differently while rotating during the scanning process because of gravity.

To measure the shifts of the projection of the metal marker in cone‐beam projections at different angular views due to OBI sagging, we developed a marker‐tracking algorithm that tracks the position of the marker (center of the metal ball) in a full sequence of nearly 650 cone‐beam projections over a full circle (360°). This marker‐tracking algorithm employs normalized cross‐correlation^(22)^ between the intensity of a template image, *T*(*x,y*), of the metal marker and an image, *I*(*x,y*), from the sequence of radiographic projections as given by Eq. (1). The origin (0,0) is considered the left side inferior corner of the imager at 0° view. (*x,y*) are the pixels of a projection with x=1,2,…,1024, and y=1,2,…,768,m=1024,n=768, representing projection dimensions, and $\bar{I}$ and $\bar{T}$ are average intensities of the image and template image, respectively. The template image is created by cropping a ROI from one of the radiographic projections that includes the metal marker. This algorithm uses both texture and shape features of the metal marker and surrounding ROI and finds the position of the point of maximal overlap and similarity between the template marker image and the radiographic projections. The position of maximal cross‐correlation, *r*(*u,v*), is assumed to represent the center of the metal marker. The error in localization of the marker is about ±0.5pixel(±0.13mm at the OBI isocenter).

(1) $$r(u,v)=\frac{\sum_{x,y=1}^{m,n}[I(x,y)-\bar{I}][T(x-u,y-v)-\bar{T}]}{\sqrt{\sum_{x,y=1}^{m,n}(I{\left(x,y)-\bar{I}\right)}^{2}\sum_{x,y=1}^{m,n}{\left(T(x-u,y-v)-\bar{T}\right)}^{2}}}$$

The shifts of the marker in *x* and *y* directions due to sagging are used as a transformation vector to map the metal marker position from various angular views for a full sequence of radiographic projections to a reference position, which was chosen to match with the center of imager (x=512 pixels and y=384pixels). The marker position shift along the x‐axis of a projection represents OBI isocenter shift in anterior‐posterior and right‐left plane using patient coordinate system. The marker shift along the y‐direction represents the superior‐inferior OBI isocenter shift. (All the directions in this work are given in patient supine head‐first coordinates.) This transformation vector, *u,v*, removes shifts in the position of the marker in the cone‐beam radiographic projections due to sagging shifts. The resultant 2D intensity map $I^{'}(x^{'},y^{'})$ of the transformed radiographic projection at a certain angular view, θ, is given by the following equation:

(2) $$I^{\prime }(x^{\prime },y^{\prime },\theta )=I(x-u,y-v,\theta )$$

The marker position in the projections shifts mainly along y‐direction and the track of these shifts can be fitted with a sinusoidal function that is a function of the view angle with a shift of about 4 mm from peak to valley, as shown in Fig. 2(b). The measured shifts along x‐directions (superior‐inferior) were small (less than 1 pixel), as shown in Fig. 2(c), where we assumed that these shifts are negligible and are not used to correct the projections. Figures 2(b) – 2(c) show outlier points on the data curve of the sagging shifts around the view angle of 90°. These points are produced by failure of the tracking algorithm to detect the shadow of the metal marker in the corresponding radiographic projections. This failure of the algorithm to detect the marker is due to the existence of shadows that may have similar intensity‐gradient features as the marker in these particular radiographic projections. Also, the step pattern of the marker shifts (Fig. 2(b)) results in small sagging shifts (within the same pixel or 0.39 mm at the imager level) and thus the shifts reported by the tracking algorithm are discretized to the same pixel. In order to resolve the previous issues with the metal marker tracking, the measured data curve of the marker position along x direction was fitted to a simple sinusoidal function according to Eq. (3). The shifts from the fitting function were used as translation vectors to remap the projections in order to eliminate the shifts due to the OBI sagging in the x direction.

(3) $$\begin{array}{rl} & u=\alpha .\cos(2\pi (\theta -\phi ))\\ & v=0 \end{array}$$

where the best fitting parameters α represents the maximum position shift of the marker along x direction, and ϕ is view angle of a projection that corresponds to maximal shift.

The transformed radiographic projections, $I^{'}$, provided by Eq. (2) were input to an image reconstruction program based on the Feldkamp back‐projection algorithm^(^ ^23^ ^,^ ^24^ ^)^ provided by the vendor (Viva, Varian Medical Systems). This technique of spatial mapping of the cone‐beam radiographic projections was applied to remove sagging shifts from both the cubical and Catphan phantoms used to evaluate mechanical stability and image quality of the OBI, respectively. Processing of 650 cone‐beam projection images to remove sagging shifts takes about 2 minutes using an in‐house developed MATLAB code (Mathworks, Inc., 3 Apple Hill Drive, Natick, MA) that runs on a PC having an Intel Core Solo Processor U1400 of 1.2 GHz and 1 GB RAM. The reconstruction of CBCT images from the processed projections takes the same time as reconstruction of original projections.

### A.4 OBI quality assurance

In addition to measuring the sagging shifts explained above, we have designed testing methods to evaluate the mechanical stability and image quality which are part of a comprehensive QA procedure for our OBI kV imaging systems. This QA procedure includes routine tests that are performed daily and monthly for the mechanical features, and quarterly for image quality. The OBI QA procedure was integrated with our clinical quality control procedures for patient treatment using IGRT setup and tumor localization techniques.

The daily QA involves mechanical testing of the accuracy of OBI isocenter to maintain accurate IGRT patient setup and localization using an orthogonal pair of kV 2D radiographic projections. Two orthogonal kV projections are acquired with the cubical phantom aligned with room lasers. The 3D shifts of the metal marker are calculated by image registration with reference images that has the metal marker aligned to radiation isocenter. The shifts should be ≤1mm in each direction. This test is performed after verification that the lasers match with radiation isocenter in our regular machine daily QA. The monthly QA consists of mechanical checking of the OBI isocenter accuracy to maintain accurate IGRT patient setup and target localization using kV 3D CBCT. Monthly CBCT isocenter stability is evaluated by the following tests of the mechanical features of the OBI: (a) sagging, (b) match of CBCT and radiation isocenter, (c) stability of rotational motion, and (d) stability of the translation motion, extension and retraction process of the arms of the imager and source. Sagging is tested by alignment of the cubical phantom with room lasers and then the projections over a full rotation of the gantry are collected and analyzed, as explained above. The match of CBCT and radiation isocenter is tested by measuring the offset of the metal marker in the center of the cubical phantom from the effective center of the CBCT volume that represents radiation isocenter. The effects of rotation on the CBCT isocenter are tested by HF and FF scans with clockwise and counterclockwise rotation of the OBI. Translational shifts are measured by performing FF and HF scans of the cubical phantom where the imager shifts off‐center for HF scans.

Image quality performance of CBCT is tested quarterly using the Catphan phantom that has various modules to test CT number uniformity, linearity, contrast, and position resolutions, as mentioned previously. This phantom is scanned on a quarterly basis using CBCT protocols that include half‐fan, full‐fan, and with and without bowtie filters in place.

## III. RESULTS

### A.1 Imager sagging

**Figure 3:**
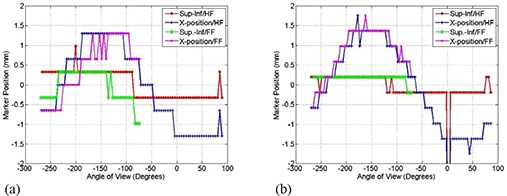
Shifts of the metal marker (a) along and perpendicular to the superior‐inferior direction in patient coordinates as a function of imaging view angle using HF and FF scans as indicated for the Trilogy machine; (b) shifts as in (a) for the second 21EX machine with a kV on‐board imager. The CBCT scans were acquired with the bowtie filters.

Figure 2(b) shows that marker shifts due to OBI sagging on one of the 2100EX linacs depends on the gantry angle with a maximum of 2 mm at about −155° . Shifts of the metal marker due to OBI sagging are independent of the scanning protocol such as HF or FF with the gantry rotated clockwise or counter‐clockwise. Figure 2(c) shows that OBI shifts in the superior‐inferior direction during the gantry rotation process are smaller than 1 pixel (0.26 mm at isocenter). The sagging shifts are produced by flexes of the supporting robotic arms of the imager and kV source due to their weights. The OBI sags mostly in its rotation plane but not in the superior‐inferior direction, which is perpendicular to gravitational force. We have measured sagging for another two Varian machines and the shifts are shown in Figs. 3(a) – 3(b). The sagging shifts vary with the view angle from one machine to another because the bearing systems of the gantry and OBI are different from one machine to the other. The sagging shifts on the Trilogy machine are the smallest and the maximal sagging was <1.5mm at angular views of −50° and 50°.

**Figure 4:**
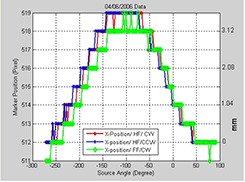
Position of the metal marker in the projections along the x‐axis as a function of view angle for the indicated CBCT scanning protocols measured seventeen months earlier of the scans in Fig. 2(b) for the first 21EX machine.

Although on a linac OBI sag remains stable and reproducible over short time periods, Fig. 4 shows that the OBI sag may vary over a longer period of time. The position of the maximum shift along x‐direction due to sag are located at a gantry angle of −105° (Fig. 4), while Fig. 2(b) shows that the maximum sagging shift measured at a time separated by about seventeen months was at −155° for the same kV OBI on one of our 21EX Varian machines. This change in sagging may be due to variation or degradation of the bearing systems of gantry rotation and the robotic arms that support the kV source and imager.

### A.2 Sagging effects on image quality

**Figure 5:**
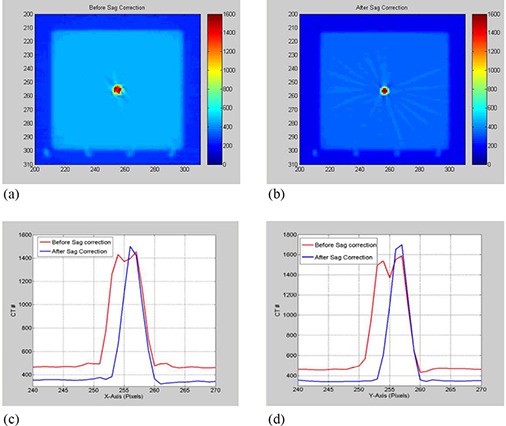
(a) and (b) show axial slices of the CBCT reconstructed from projections before and after correcting sagging shifts, respectively; (c) and (d) show line profiles from the CBCT slices through the center of the metal marker along X‐ and Y‐axis, respectively.

Figures 5(a) and 5(b) show that OBI sagging shifts induce a spread out in the shadow of the metal ball in CBCT of the cubical phantom reconstructed from projections without sag correction. Isocenter shifts as large as 1 mm anterior‐posterior and 1 mm lateral of the center of the metal marker are produced in the CBCT profiles (Figs. 5(c) and 5(d)), reconstructed from projections with and without corrections of sagging shifts along x‐axis and y‐axis, respectively. Figures 5(c) and 5(d) show that CBCT numbers decrease in the region of the shadow of the metal ball, while background CBCT numbers increase in the surrounding area due to blurring artifacts caused by sagging.

**Figure 6:**
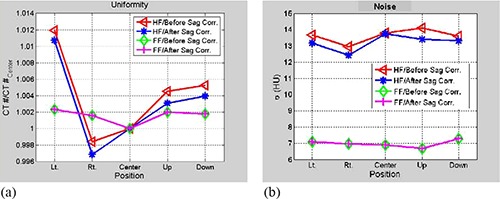
Data curves show (a) average CT number over ROI's cropped from the different places from a slice of uniformity: left, right, center anterior and posterior, before and after correction of imager sagging effects for half‐fan (HF) and full‐fan (FF) scans as indicated; (b) the corresponding standard deviation in HU units from the ROI's in (a) and scans; CBCT numbers increase by up to 3.5% in HF and about 1% in FF CBCT due to OBI sagging in the radiographic projections.

**Figure 7:**
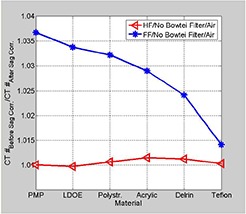
Data curves show the ratio of average CT numbers for ROI's cropped from a linearity slice with the materials indicated for HF and FF scans before and after correction for sag effects.

**Figure 8:**
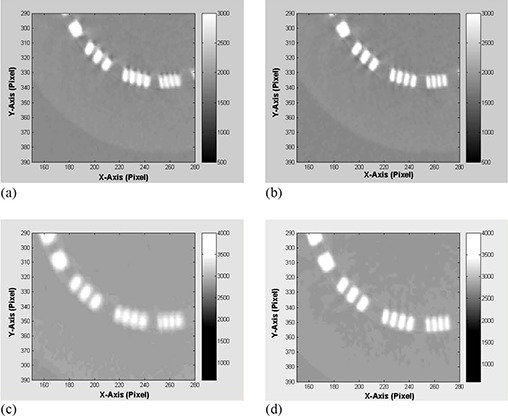
(a) and (b) show comparison between CBCT position resolution slices reconstructed from projections before and after OBI sag correction for HF scan; (c) and (d) are comparisons of position resolution for FF scans.

Figure 6(a) shows that the uniformity of CBCT as determined by CT numbers improves in HF scan by correcting sagging prior to reconstruction. The standard deviation as shown in Fig. 6(b) decreases in CBCT reconstructed from sagging‐corrected projections using HF scans. However, for FF scans, the uniformity and standard deviation in CBCT remain unchanged before and after sagging correction. CBCT numbers increase by up to 3.5% in HF and about 1% in FF CBCT due to OBI sagging in the radiographic projections, as shown in Fig. 7. The position resolution in CBCT improves in both HF and FF scans (Figs. 8(b) and 8(d)), with OBI sagging correction compared to those without correction (Figs. 8(a) and 8(c)). The geometrical integrity of the reconstructed CBCT slices for the QA phantom was tested before and after sagging correction. For example, the spatial distance between two markers was about 5 cm before and after sagging correction. However, after sag correction, the markers were less blurred and more localized. Our method of sag correction did not cause spatial distortion in the features of the QA phantom. Figures 5 and 8 show how blurring is reduced in the metal marker and the position resolution patterns while the geometrical integrity of the pattern is maintained, respectively.

### A.3 Mechanical stability

**Figure 9:**
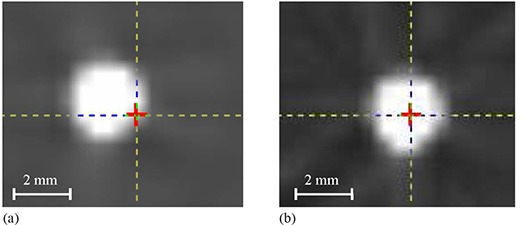
Crosshair represents position of OBI isocenter created in the center of the effective volume of the cone‐beam scan by the image registration program, while the center of the metal marker represents radiation isocenter for a full‐fan CBCT of the cubical phantom (a) before and (b) after sag correction in cone‐beam projections.

Figure 9(a) shows that the crosshair produced automatically in the center of the CBCT volume by the image registration program representing the OBI isocenter does not match with the center of the metal marker. The marker is shifted about 1.1 mm and 0.9 mm towards patient right and anterior from the isocenter of CBCT reconstructed from projections without correction for sagging, respectively (similar to the shifts in Fig. 4). The crosshair matches better with the center of the metal marker (Fig. 9(b)) in CBCT reconstructed from projections with sag correction where the marker is projected back to the center of the image (x=512pixels,y=384pixels).

**Figure 10:**
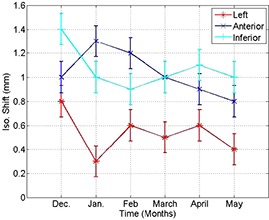
Systematic shifts of CBCT and radiation isocenters in the different directions (anterior, left and inferior in patient supine head‐first coordinates) over 6‐month time period.

The data in the second row of Table 1 show systematic shifts between CBCT isocenter and the radiation isocenter with values of 1.4 mm superior, and about 1 mm anterior and 0.8 mm left in patient coordinates on one 21EX machine using a HF scan. Gantry rotational motion causes small shifts of the position of CBCT isocenter of 0.2 mm and less in all directions when the gantry is rotated counterclockwise relative to clockwise using HF scans. Translation motion of the imager to perform HF and FF scans produces isocenter shifts in the range of 0.3–0.8 mm, as indicated in Table 1. The localization accuracy of the metal marker in CBCT is about 0.1 mm. This is limited by the smallest measurable shifts by the image registration program and the machining accuracy of the cubical phantom. Based on OBI sagging shifts shown in Figs. 2, 3 and 4, the systematic errors in matching CBCT and radiation isocenters in a plane formed by anterior‐posterior and right‐left patient directions are produced from cumulative sagging shifts that are introduced in the projections during OBI rotation. The localization of the OBI isocenter in the superior‐inferior direction depends on the reproducibility of the position of the robotic arms in the extension process before scanning. The systematic errors in the OBI isocenter due to gantry rotation result from small differences in OBI sagging when the gantry is rotating clockwise versus counter‐clockwise. Limitation of the reproducibility of the position of the OBI robotic arms and OBI sagging produces systematic shifts of CBCT isocenter in HF and FF scans. The systematic errors in the position of the OBI isocenter relative to the radiation isocenter shown in Fig. 10 persist in the same direction, and show small variation of about 0.5 mm over a time period of 6 months.

**Table 1:** Typical shifts of OBI isocenter relative to radiation isocenter in patient coordinates measured from the monthly mechanical CBCT QA.

*Isocenter Position*	*Lateral (mm)*	*Vertical (mm)*	*Longitudinal (mm)*
CBCT Radiation Isocenter ^a^	**0.8**	**1.0**	**1.4**
Isocenter Shifts from Rotational Motion^b^	**0.1**	**0.1**	**0.2**
Isocenter Shifts from Translational Motion^c^	**0.5**	**0.3**	**0.8**

^a^ The second row represents systematic offset between CBCT and radiation isocenters in the different directions: lateral, vertical and longitudinal, using HF scan.

^b^ The third row shows CBCT isocenter shifts due to rotational motion of the gantry counterclockwise relative to a scan done clockwise using HF scan.

^c^ The fourth row shows shifts of the CBCT isocenter due to the translation motion of the imager to perform HF and FF.

The error in the measured shifts is ±0.13mm.

## IV. DISCUSSION

OBI sagging induces image shifts at various gantry angles while it is rotating during scanning due to the weight of the kV source and imager, non‐rigidity of the supporting arms, and small instability of the bearing system. These shifts are different from the OBI and radiation isocenter stable offset which results from limitations in the adjustment and fine‐tuning of the mechanical setup of both systems. In this work, we found that the position of imaging isocenter in 2D projections shifts due to sagging, and it depends on the gantry angle, with a maximal shift of about 4 mm from peak to valley on one of our linacs. Further, sagging produces systematic isocenter positioning errors, spatial distortion, and degradation of the image quality in CBCT reconstructed from projections that are not corrected for sagging. These shifts propagate into about 1 mm posterior and 1 mm left systematic isocenter shifts in volumatric CBCT on one of our 21EX Varian machines. The technique developed in this work, in which sagging shifts are measured and the projections are mapped to the right reference position prior to image reconstruction, removes the isocenter systematic shifts, reduces spatial distortion, and improves image quality in CBCT.

The systematic shifts in OBI isocenter in both 2D orthogonal projections and 3D CBCT images due to sagging as reported in this work limits the use of OBI imaging as a tool for accurate patient setup and tumor localization. Patients treated with high‐dose gradient IMRT or stereotactic radiation therapy require strict accuracy of patient setup and tumor localization within a couple of mm. Though IGRT procedures based on 2D radiographs or 3D CBCT obtained before or during patient treatment can provide more accurate patient setup and tumor localization using internal patient anatomy, the systematic positioning errors due to sagging must be considered in order to improve accuracy of patient setup and tumor localization.

We used two methods to correct systematic positioning errors of CBCT isocenter: (a) remapping of the cone‐beam radiographic projections to compensate for sagging effects due to gravity prior to CBCT reconstruction, or (b) application of reverse shifts to the isocenter in the different directions in CBCT in order to correct sagging shifts. The first technique corrects sagging in the projections prior to reconstruction. In the second technique, cumulative isocenter shifts due to sagging in the reconstructed CBCT are measured and corrected in image registration process for IGRT patient setup and tumor localization. Besides compensating for CBCT isocenter shifts, the first technique reduces spatial distortion, blurring artifacts, shifts of CT number, and lack of uniformity. Blurring artifacts in CBCT due to OBI sag and spatial distortion produce enlarged volumes for the target or critical structures. These structures outlined using blurred CBCT without correction of OBI sagging effects may introduce dose shortage of the target or overdosing of critical structures and normal tissue. Thus, OBI sagging shifts must be considered in order to achieve more accurate patient setup and tumor localization. This is especially important in special procedures such as IMRT or stereotactic radiation therapy where high precision and accuracy in patient setup and tumor localization affect the outcome of treatment in terms of tumor coverage and sparing of critical structures.

OBI sagging shifts and systematic errors in the positioning of CBCT isocenter may change with time as the bearing system that supports the kV source and imager becomes older. Therefore, sagging shifts need to be frequently monitored and corrected. Image registration parameters used to correct systematic isocenter shifts due to OBI sagging used in clinical IGRT from 2D and 3D imaging must be updated as isocenter shifts due to sagging change. In addition, in patient setup and tumor localization using internal patient anatomy, the image quality of CBCT is more vulnerable to degradation effects such as OBI sagging, scatter radiation^(^ ^25^ ^,^ ^26^ ^)^ and reconstruction artifacts from metal objects^(19)^ than conventional fan‐beam CT. Therefore, the OBI needs more frequent monitoring and maintenance procedures. In this work, we introduced a clinical quality assurance procedure to monitor and maintain mechanical stability and image quality of our kV OBI. We recommend performance of daily and monthly QA tests for the mechanical sagging, and quarterly image quality QA tests. Correction techniques for systematic errors of CBCT isocenter are required to establish clinically acceptable standards. In order to obtain accurate localization of the isocenter, in‐house or commercial image registration programs should have integrated tools to account for systematic shifts due to sagging.

## V. CONCLUSIONS

Sagging of the kV OBI produces systematic shifts in 2D images and 3D CBCT that cause mismatch between OBI and radiation isocenter. Correction of sagging should be considered in order to produce more accurate patient setup and tumor localization using kV on‐board imaging. Sagging causes image artifacts such as spatial distortion, blurring artifacts, degradation of soft tissue contrast, and position resolution in CBCT. Further, OBI sagging changes CT numbers, which invalidates corresponding electron density for dose calculation. The technique developed in this work to correct sagging shifts by preprocessing of cone‐beam radiographic projections prior to reconstruction removes systematic errors in the CBCT isocenter and improves image quality. Sagging correction reduces spatial distortion and blurring artifacts, improves contrast and position resolutions, and increases uniformity and linearity of CBCT numbers. QA procedures are required to test and monitor variation in OBI mechanical accuracy and image quality and to maintain high clinical standards of IGRT procedures for more accurate patient setup and tumor localization based on internal patient anatomy using 2D and 3D kV on‐board imaging.
